# IL-10 Production Is Critical for Sustaining the Expansion of CD5^+^ B and NKT Cells and Restraining Autoantibody Production in Congenic Lupus-Prone Mice

**DOI:** 10.1371/journal.pone.0150515

**Published:** 2016-03-10

**Authors:** Yuriy Baglaenko, Kieran P. Manion, Nan-Hua Chang, Eric Gracey, Christina Loh, Joan E. Wither

**Affiliations:** 1 Department of Genetics and Development, Krembil Research Institute, University Health Network, Toronto, Ontario, Canada; 2 Department of Immunology, University of Toronto, Toronto, Ontario, Canada; 3 Department of Medicine, University of Toronto, Toronto, Ontario, Canada; Instituto Nacional de Ciencias Medicas y Nutricion Salvador Zubiran, MEXICO

## Abstract

The development and progression of systemic lupus erythematosus is mediated by the complex interaction of genetic and environmental factors. To decipher the genetics that contribute to pathogenesis and the production of pathogenic autoantibodies, our lab has focused on the generation of congenic lupus-prone mice derived from the New Zealand Black (NZB) strain. Previous work has shown that an NZB-derived chromosome 4 interval spanning 32 to 151 Mb led to expansion of CD5^+^ B and Natural Killer T (NKT) cells, and could suppress autoimmunity when crossed with a lupus-prone mouse strain. Subsequently, it was shown that CD5^+^ B cells but not NKT cells derived from these mice could suppress the development of pro-inflammatory T cells. In this paper, we aimed to further resolve the genetics that leads to expansion of these two innate-like populations through the creation of additional sub-congenic mice and to characterize the role of IL-10 in the suppression of autoimmunity through the generation of IL-10 knockout mice. We show that expansion of CD5^+^ B cells and NKT cells localizes to a chromosome 4 interval spanning 91 to 123 Mb, which is distinct from the region that mediates the majority of the suppressive phenotype. We also demonstrate that IL-10 is critical to restraining autoantibody production and surprisingly plays a vital role in supporting the expansion of innate-like populations.

## Introduction

Systemic lupus erythematosus (SLE) is a multifactorial autoimmune disorder characterized by the production of pathogenic anti-nuclear antibodies (ANAs). A combination of genetic and environmental factors interacts to initiate and exacerbate disease in patients with SLE. To decipher the genetics of SLE initiation and progression, studies in our lab and others have focused on generating congenic mice where susceptibility or suppressor loci from lupus-prone mouse strains can be examined in isolation [1].

The prototypic murine model of SLE is the F1 cross between the New Zealand Black and New Zealand White (NZB/W F1) mouse strains, which develop high titer ANAs and fatal renal disease by 8 months of age. Since NZB/W F1 mice have a mixed genetic background, homozygous derivatives were created to map the genetic defects associated with disease. One of these derivatives, the NZM2410 mouse strain, was used to identify three major susceptibility loci on chromosomes 1, 4, and 7 named *Sle1*, *Sle2*, *and Sle3*, respectively [2–4]. Although the *Sle1* and *Sle3* susceptibility loci were derived from the NZW parent, *Sle2* contained a mixture of NZB and NZW genetic material, with the NZB interval extending from 100 to 128 Mb.

Studies from our lab have focused on investigating how New Zealand Black (NZB) genes on chromosomes (c) 1, 4, and 13 influence immune function. Initial work on B6 mice with an introgressed NZB c4 interval extending from 32 to 151 Mb, denoted B6.NZBc4, identified an expansion of two innate-like populations, B1a cells and Natural Killer T cells (NKT), in the absence of autoantibody production or renal disease [5]. As previous mapping studies had suggested the presence of a lupus-susceptibility gene within this interval, we anticipated that crossing this interval onto the lupus-prone B6.NZBc1 congenic background would lead to augmented autoimmune disease. However, this cross resulted in suppression of disease with reduced autoantibody levels and kidney damage as compared to mice with the NZB c1 interval alone [6].

In a recent follow-up publication, we investigated the immune mechanism leading to this suppression and ruled out a regulatory role for the expanded NKT cell population by creating CD1d knockout B6.NZBc1c4 bicongenic mice. Instead, a possible regulatory role for the expanded splenic CD5^+^ B cell compartment was identified [7]. Given the recent interest in regulatory B cells, we hypothesized that IL-10 production by CD5^+^ B cells was critical to suppression in our lupus-prone mice.

Over the last decade, research has highlighted the suppressive role of IL-10 producing regulatory B cells in various autoimmune models ranging from collagen-induced arthritis to experimental autoimmune encephalomyelitis [8–10]. Pertinent to our studies, IL-10 producing regulatory B cells have also been identified to play a suppressive role in several mouse models of SLE [11–13]. In the NZB/W F1 model, depletion of B cells early in disease resulted in a loss of regulatory B cells and an accelerated phenotype [11]. In the MRL/lpr mice model, which have a defect in Fas and are therefore prone to autoimmunity, induction of regulatory B cells through anti-CD40 stimulation and subsequent adoptive transfer was shown to have an IL-10 dependent protective effect [14]. Disease modulating IL-10-producing B cells have been characterized in numerous B cell compartments ranging from typical B1 and marginal zone (MZ) B cells to specific sub-populations such as transitional 2-marginal zone precursors and CD1d^hi^CD5^+^ B10 cells [8,15]. Although their ontogeny and phenotypic characteristics are still not entirely known, through use of knockout animals and blocking antibodies, IL-10 has been shown to play a central role in the suppressive function of these cells [9,16].

IL-10 is a pleiotropic cytokine produced by a number of leukocyte populations that impacts on immune regulation and tissue homeostasis [17,18]. While its expression by regulatory B cell populations suggests that it may play a predominantly suppressive role in SLE, the evidence supporting this is contradictory. Studies of murine models of lupus have identified both pathogenic and suppressive roles for IL-10 in disease. In the NZB/W F1 mouse model, administration of blocking IL-10 antibodies reduced disease severity while prolonged treatment with recombinant IL-10 accelerated disease [19]. In contrast to these studies, knockout of IL-10 exacerbated disease and administration of recombinant protein lowered the levels of autoantibodies in MRL/lpr mice [20]. However, a B cell specific IL-10 knockout bred onto the MRL/lpr background had no effect on the progression or severity of SLE [21]. In support of a suppressive role for IL-10 in SLE, triple-congenic B6.*Sle1*.*Sle2*.*Sl*e*3* mice that were forced to produce low levels of IL-10 by introduction of a viral vector had delayed autoantibody production and decreased nephritis [22].

In this study, we have used a combination of NZB c4 sub-congenic mouse strains and IL-10 knockout mice to further investigate the immune mechanisms leading to suppression of autoimmune disease and expansion of CD5^+^ B cells and NKT cells in B6.NZBc4 mice. We show that although suppression of autoimmunity and expansion of these cell subsets localize to different regions on NZB c4, both are dependent upon IL-10. To our knowledge, this is the first study to identify a possible link between global IL-10 production and the survival and/or expansion of CD5^+^ B cells and NKT cells, suggesting a possible role for IL-10 in supporting suppressive immune populations.

## Results

### The expansion of innate-like lymphocytes localizes to the New Zealand Black chromosome 4 interval spanning 91 to 123 Mb

**Fig 1:**
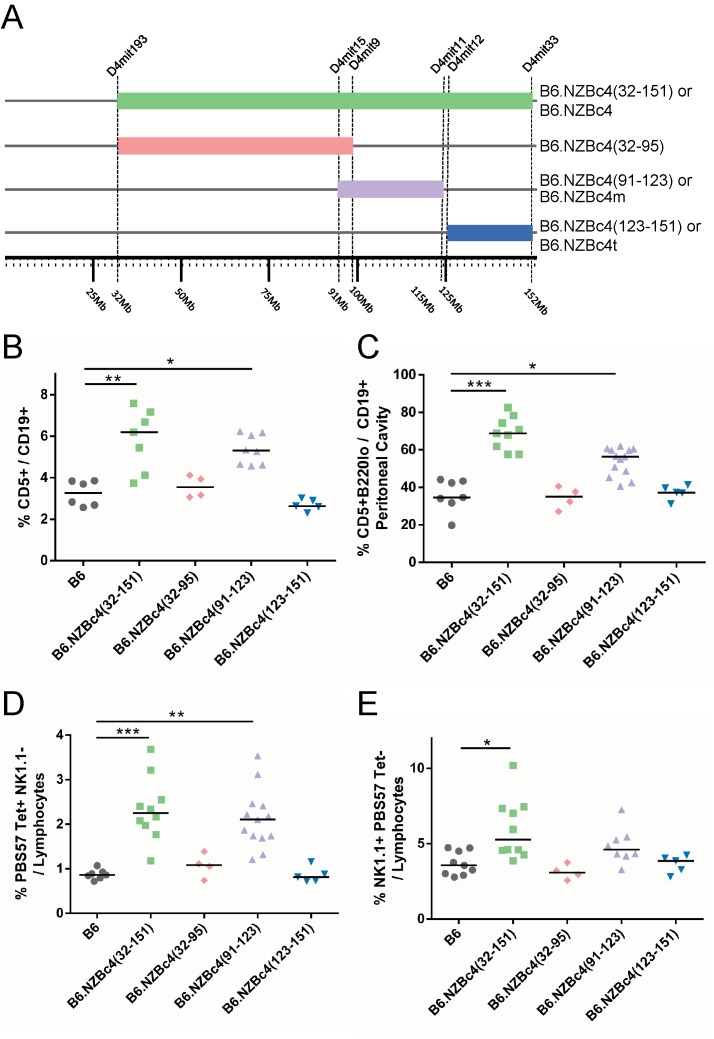
The expansion of peritoneal B1a B cells, splenic CD5^+^ B cells, and NKT cells localizes to an NZB-derived interval spanning 91 to 123 Mb on chromosome 4. (A) Figure illustrating the NZB chromosome 4 congenic strains used in these studies. D4Mit markers demarcate the known boundaries of introgression. Splenic and peritoneal immune cell frequencies were measured by flow cytometry from 4 month old mice. (B) Splenic CD5^+^ B cells were measured based on FSC, SSC, CD19, and CD5 staining. (C) Frequencies of peritoneal cells were identified by granularity and size and gated as CD19^+^ and CD5^+^. (D) Splenic NKT cells were gated based on size and granularity using FSC, SSC, and gated as PBS57 Tetramer^+^ NK1.1^-^. (E) Splenic NK cells were measured based on FSC, SSC, and gated as NK1.1^+^ and PBS57 Tetramer^-^. Each point represents a single mouse, with the lines indicating the median of each group. Statistical analyses were carried out using a Kruskal-Wallis test with select Dunn multiple comparison posttests to control B6 mice. * P < 0.05, ** P < 0.01, ***P <0.001.

As outlined previously, introgression of an NZB c4 interval spanning 32 to 151 Mb onto the B6 genetic background led to an increase in CD5^+^ B cells and NKT cells [5]. In an effort to better characterize and delineate the genes involved in these expansions and disease suppression, sub-congenic mice were bred with shorter NZB c4 intervals (Fig 1A). Innate-like immune populations were then characterized by flow cytometry within the peritoneal and splenic compartments of 4 month old full-length and sub-congenic B6.NZBc4 mice. As reported previously [5], B6.NZBc4 mice have a slight expansion of total peritoneal cells, but not total splenocytes (S1 Fig).

In sub-congenic mice, expansion of splenic CD5^+^ B cells (Fig 1B), peritoneal B1a cells (Fig 1C), and splenic NKT cells (Fig 1D) was only seen in mice with the middle sub-congenic interval B6.NZBc4(91–123), termed B6.NZBc4m for simplicity, which largely recapitulated the expansion seen for the full-length B6.NZBc4 mice. Although there was a trend to decreased levels of peritoneal B1a cells in B6.NZBc4m as compared to B6.NZBc4 mice, this did not achieve statistical significance. However, we cannot rule out a contribution of genes in the telomeric B6.NZBc4(123–151) interval, henceforth termed B6.NZBc4t, to this phenotype as we have previously noted minor increases in the peritoneal B1a cell population in these mice. Surprisingly, the increase of NK1.1^+^ cells (Fig 1E) seen in B6.NZBc4 mice could not be recapitulated with any single NZB interval, suggesting that a combination of genes is required to promote this phenotype.

### The expansion of B1a and NKT cells has minimal impact on the suppression of autoimmunity in B6.NZBc1c4t mice

**Fig 2:**
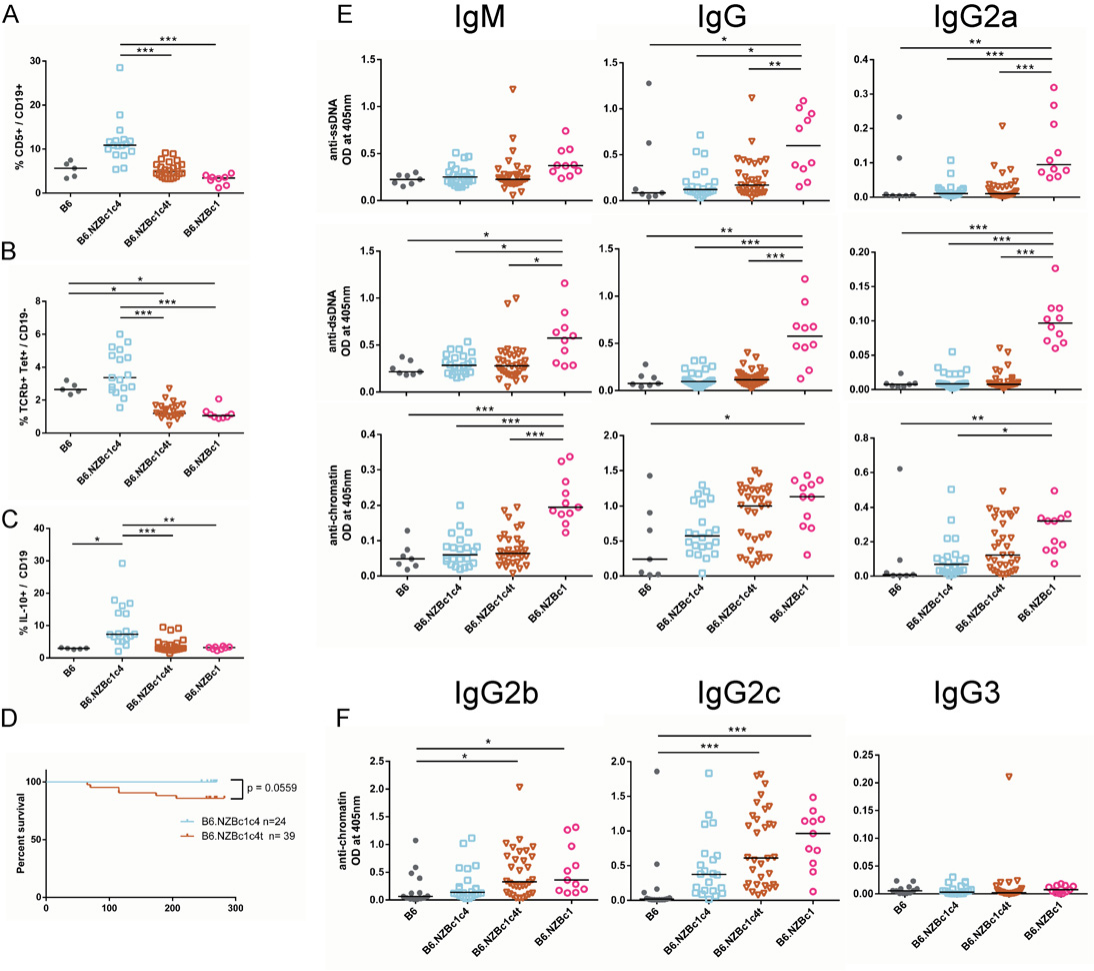
Suppression of anti-ssDNA and–dsDNA autoantibody production in bicongenic B6.NZBc1c4(123–151) mice in the absence of either CD5^+^ B cells or splenic NKT cell expansion. Frequencies of splenic CD5^+^ B cells (A), NKT cells (B), and IL-10 competent B cells (C) were measured by flow cytometry in 8 to 10 month old mice. (D) Survival curves of aged B6.NZBc1c4 and B6.NZBc1c4t mice show a trend (p = 0.0559) towards increased mortality in B6.NZBc1c4t mice. Levels of anti-ssDNA, -dsDNA, and–chromatin IgM, IgG and IgG2a (E) were measured by antigen-specific ELISA. To define the high levels of anti-chromatin autoantibodies in B6.NZBc1c4(123–151) mice, IgG2b, IgG2b, and IgG3 anti-chromatin antibodies were measured by ELISA (F). Each point represents a single mouse, with the lines for each group representing the median. Statistical analyses were carried out using a Kruskal-Wallis test with Dunn multiple comparison posttests between all groups. * P < 0.05, ** P < 0.01, ***P < 0.001.

We have previously shown that crossing the full-length (32 to 151 Mb) NZB c4 interval onto the lupus-prone B6.NZBc1 background leads to suppression of autoantibody production and renal disease, and have provided evidence through adoptive transfer experiments that this was mediated by a suppressive effect of NZB c4 CD5^+^ B cells [6,7]. This led us to hypothesize that the suppression was the result of the expansion of CD5^+^ B cells in B6.NZBc4 mice. To address this hypothesis, bicongenic mice were generated with the telomeric NZB c4 interval spanning 123 to 151 Mb (c4t) and contrasted to bicongenic mice with the full-length NZB c4 interval. Mice were aged to 8 months of age and analyzed for autoantibody production by antigen-specific ELISA. In contrast to bicongenic mice with the full-length interval (B6.NZBc1c4), bicongenic mice with the telomeric interval (B6.NZBc1c4t) had minimal, non-significant increases in the proportions of splenic CD5^+^ B cells, NKT cells, and IL-10-producing B cells as compared to B6.NZBc1 mice (Fig 2A, 2B and 2C). Although we observed a trend (p = 0.0559) towards increased mortality in B6.NZBc14t mice suggesting a slight decrease in suppression (Fig 2D), production of IgM anti-dsDNA and–chromatin, together with IgG anti-ssDNA and–dsDNA antibodies was equivalently suppressed in both bicongenic mouse strains as compared to B6.NZBc1 mice (Fig 2E). However, some small differences in the ability of the two intervals to suppress IgG anti-chromatin antibody production were seen. There was a trend to decreased suppression of IgG anti-chromatin antibody production with the telomeric NZB c4 interval, particularly for the IgG2b and IgG2c subclasses (Fig 2F). Thus, the genetic locus mediating suppression of autoimmunity in bicongenic mice is distinct from that leading to the marked expansion of CD5^+^ and NKT cells on NZB c4, with the exception of a minor additive effect on IgG anti-chromatin antibody production.

### Knockout of IL-10 is sufficient to breach tolerance in B6.NZBc4 mice

Previous mapping studies suggested the presence of a lupus-susceptibility locus on NZB c4, however B6.NZBc4 mice do not produce anti-nuclear autoantibodies [5]. Since we had previously shown that the CD5^+^ B cells in B6.NZBc4 mice have a regulatory function and produce IL-10, we questioned whether knockout of IL-10 could uncover autoimmunity [7]. Knockout mice were produced by backcrossing an IL-10 gene deletion onto both the B6.NZBc4 and B6.NZBc4m backgrounds, with the efficacy of the knockout being confirmed by measuring IL-10 production after stimulation with LPS, PMA, and ionomycin in the presence of monensin (S2 Fig).

**Fig 3:**
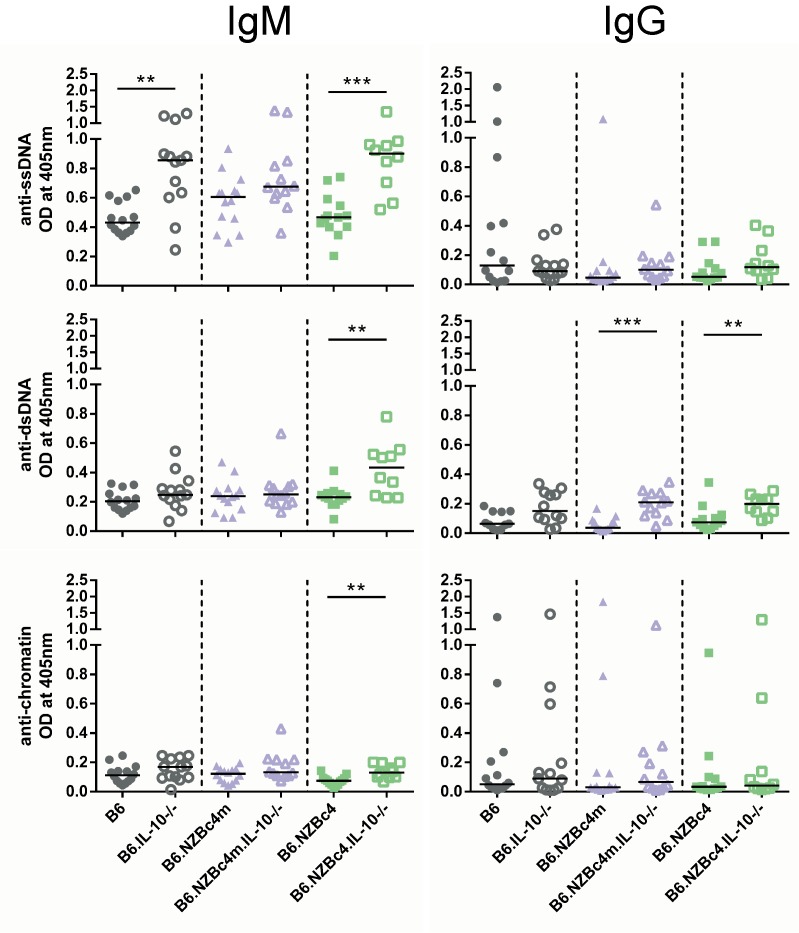
Knockout of IL-10 in B6.NZBc4 but not B6.NZBc4m mice results in a breach of tolerance to ssDNA, dsDNA, and chromatin. Levels of anti-ssDNA, -dsDNA, and–chromatin antibodies were measured in 4 month old mice by ELISA. Each point represents a single mouse, with the lines for each group representing the median. Statistical analyses were carried out using a Mann-Whitney *U* test between homozygous and IL-10 knockout animals of the same genetic background. * P < 0.05, ** P < 0.01.

Supporting our previous findings suggesting that CD5^+^ IL-10-producing B cells are suppressive, IL-10 knockout resulted in increased production of anti-ssDNA, -dsDNA, and -chromatin IgM autoantibodies in B6.NZBc4 mice, as measured by antigen-specific ELISA on serum from 4 month old mice (Fig 3). B6.IL-10-/- mice also exhibited an increase in IgM anti-ssDNA autoantibodies, but not other nuclear antigens. Both B6.NZBc4m and B6.NZBc4 mice had increased levels of anti-dsDNA IgG autoantibodies (Fig 3). However, there were no changes in the levels of IgG1 or IgG2a autoantibodies (S3 Fig), suggesting a largely T cell independent breach of tolerance. This finding is in line with our other observations, which have shown that T and dendritic cell defects from genetic loci on NZB c1 are required to promote SLE and IgG anti-nuclear antibody production in the NZB mouse strain [23]. From this data, it is evident that only B6.NZBc4 mice had a consistent breach in autoantibody production, suggesting the presence of additional NZB-derived c4 loci that can exacerbate autoimmunity.

### Expansion of splenic CD5^+^ B cells and peritoneal B1a cells is reliant on IL-10

Since knockout of IL-10 led to enhanced autoantibody production, we sought to determine whether this resulted solely from a lack of IL-10 production or whether other immunologic changes might contribute to this breach of tolerance. As we had previously shown that adoptive transfer of NZB c4 CD5^+^ B cells led to enhanced suppression of T cell pro-inflammatory cytokine production in B6.NZBc1 autoimmune mice, as compared to transfer of CD5^-^ cells, we examined whether introduction of the IL-10 knockout onto the B6.NZBc4 background affected this population [7].

**Fig 4:**
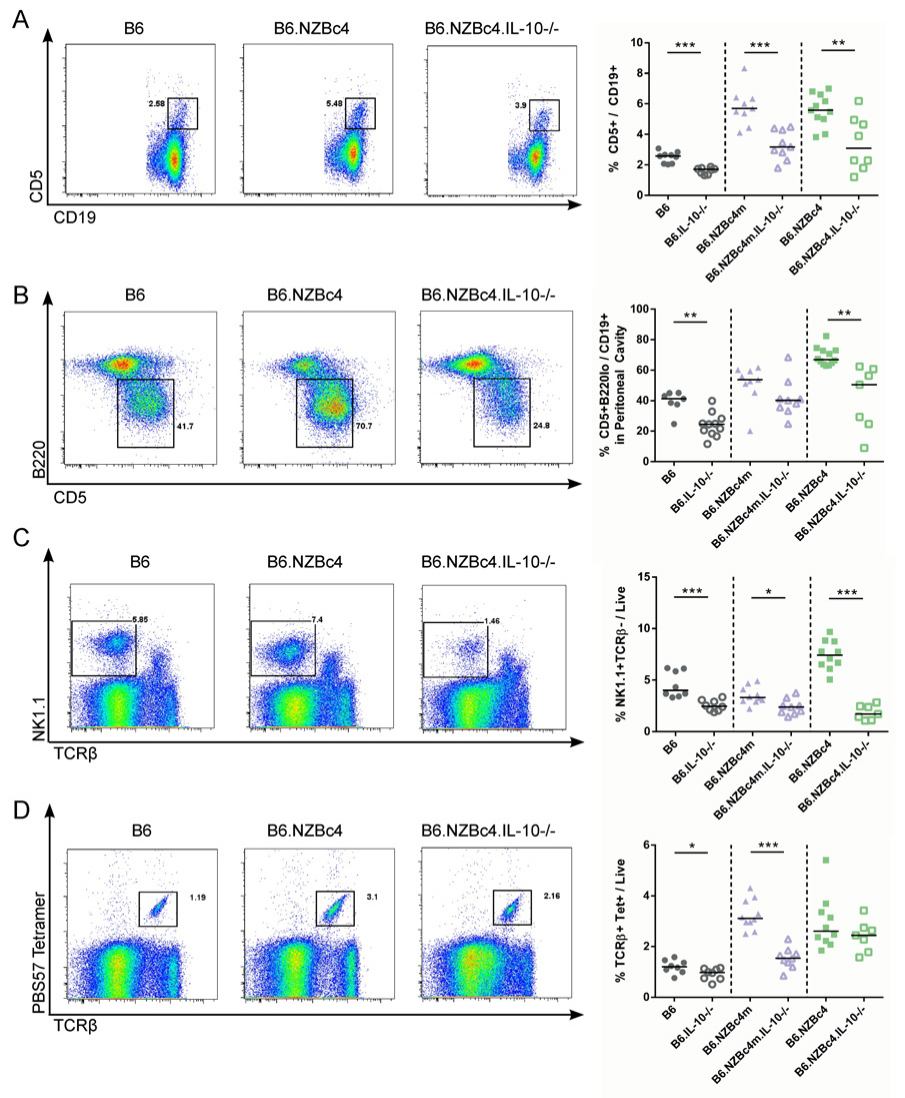
The expansion of CD5^+^ B cells, NK and NKT cells is impacted by the loss of IL-10. The frequency of splenic and peritoneal cells was measured in 4 month old mice by flow cytometry. Representative gating and results for splenic CD5^+^ B cell (A), peritoneal CD5^+^ B cell (B), NK cell (C) and NKT cell frequencies (D). Each point represents a single mouse, with the lines for each group representing the median. Statistical analyses were carried out using a Mann-Whitney *U* test between homozygous and IL-10 knockout animals of the same genetic background. * P < 0.05, ** P < 0.01, ***P <0.001.

As shown in Fig 4, knockout of IL-10 significantly reduced the frequency of CD5^+^ B cells in the spleen and peritoneum of both control and NZB c4 congenic mouse strains. However, in the IL-10 knockout NZB c4 mouse strains, the proportion of these cells remained somewhat expanded as compared to that seen in B6 IL-10 knockout mice.

The loss of these two CD5^+^ populations prompted us to investigate whether expansion of other innate-like immune populations was also altered in our congenic IL-10 knockout mice. As has been previously shown by others, since proliferation of NK cells is reliant on IL-10, knockout of this cytokine significantly reduced the proportion of these cells in all mouse strains examined (Fig 4C) [24,25]. Surprisingly and previously unreported, the expansion of NKT cells was also dependent on IL-10 and was significantly reduced in B6 and B6.NZBc4m mice (Fig 4D).

Given the relationship between regulatory CD5^+^ B cells and T regulatory cells [26], we also assessed the frequency of these cells in B6.NZBc4m mice. However, the loss of CD5^+^ B cells had no impact on T regulatory cells in IL-10 knockout B6.NZBc4m mice (S4 Fig).

**Fig 5:**
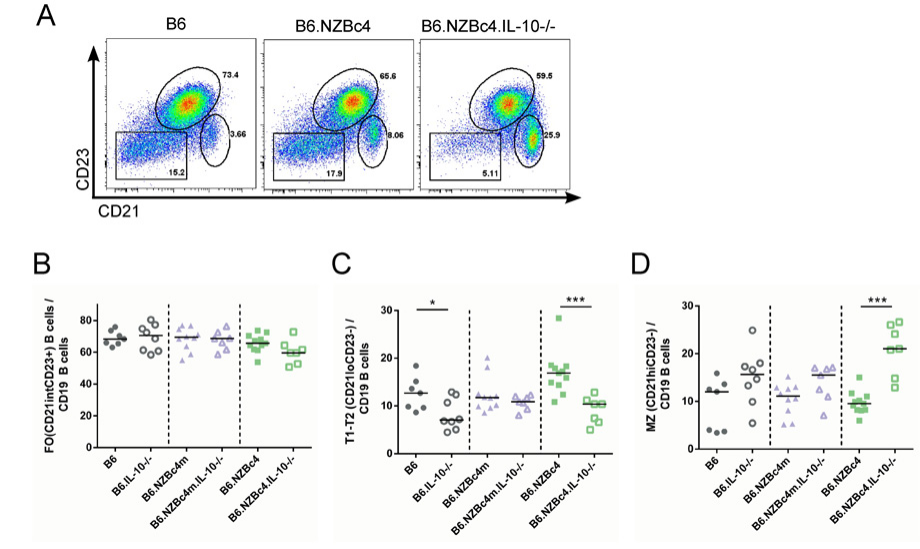
Knockout of IL-10 in full-length B6.NZBc4 but not B6.NZBc4m mice resulted in a loss of transitional B cells and an expansion of marginal zone B cells. (A) Representative gating of transitional (CD21^lo^CD23^-^), follicular (CD21^int^CD23^+^), and marginal zone/marginal zone precursor (CD21^hi^CD23^-^) B cells from 4 month old mice. Frequencies of splenic B cell subsets were measured by flow cytometry. (B,C,D) The frequency of follicular, transitional and marginal zone B cells, respectively, was measured by flow cytometry as gated in (A). Each point represents a single mouse, with the lines for each group representing the median. Statistical analyses were carried out using a Mann-Whitney *U* test between homozygous and IL-10 knockout animals of the same genetic background. * P < 0.05, ** P < 0.01, ***P < 0.001.

Finally, we assessed whether the IL-10 knockout affected the frequency of splenic B cell populations. Importantly, IL-10 did not alter the number of splenocytes or proportion of B cells between mouse strains (S1 Fig). As shown in Fig 5, there was a significantly reduced frequency of transitional B cells (CD21^lo^CD23^-^), and expanded proportion of marginal zone B cells (CD21^hi^CD23^-^) in B6.NZBc4 IL-10 knockout mice. In contrast, the frequency of follicular B cells (CD21^int^CD23^+^) was unaffected in any of the mouse strains. This data raises the possibility that an increase in marginal zone B cells may contribute to the production of IgM autoantibodies in B6 and B6.NZBc4 mice, as other groups have demonstrated that marginal zone B cells can produce IgM in the absence of T cell help [27]

### Production of IL-10 is critical to peritoneal B cell survival

Previously published work has localized the expansion of peritoneal CD5^+^ B cells in NZM2410 mice to a region overlapping with the NZB c4m interval and shown that this is due to defective p18 expression [28,29]. Loss of p18, a cell cycle inhibitor, results in increased turnover of peritoneal B1a cells.

**Fig 6:**
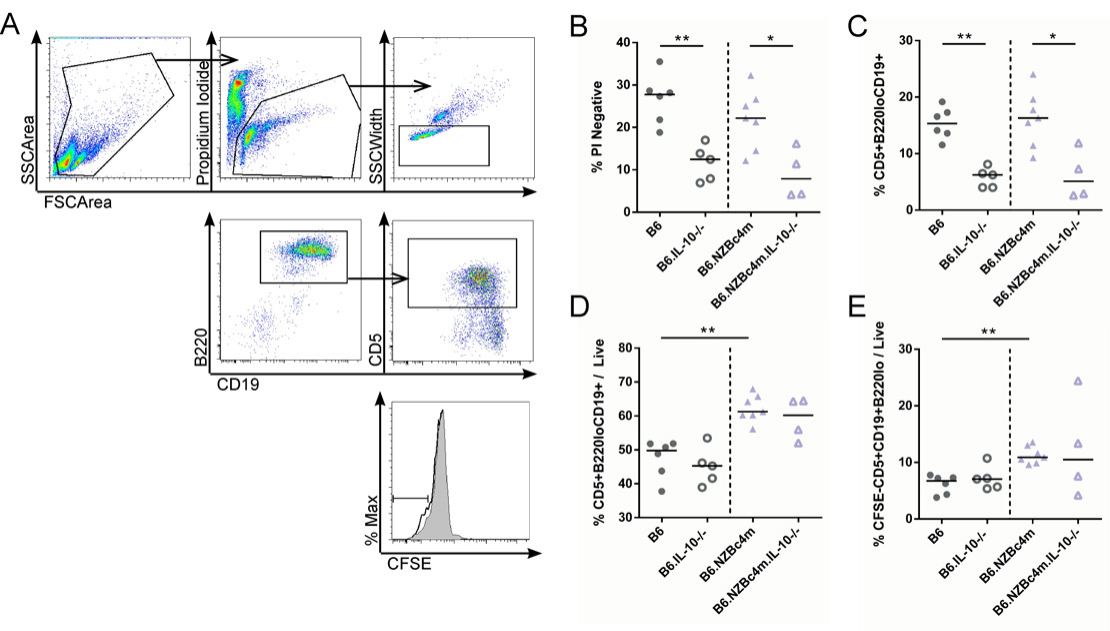
IL-10 is required for cell survival but has no impact on the proliferation of peritoneal B1a cells. Peritoneal B cells were harvested and stained with CFSE as described in the Materials and Methods. Cells were cultured without stimulation for 5 days and homeostatic turnover measured by flow cytometry. (A) Representative gating of live (PI^-^, Doublet-excluded), B1a (B220^lo^CD19^+^CD5^+^) and proliferating (CFSE^lo^) cells is shown. Frequency of all live cells (B) and proportion of B1a cells within the live population (C) were measured by flow cytometry as a proportion of total events. The frequency of B1a cells (D) and proliferated B1a cells (E) as a proportion of live cells was measured by flow cytometry. Each point represents a single mouse, with the lines for each group representing the median. Statistical analyses were carried out using a Mann-Whitney *U* test between homozygous and IL-10 knockout animals of the same genetic background. * P < 0.05, ** P < 0.01.

Thus, to further explore the underlying mechanism leading to the reduction in CD5^+^ B cells in IL-10 knockout B6 and NZB c4m congenic mice, rested peritoneal lavage cells from B6 and B6.NZBc4m mice were stained with CFSE and cultured for 5 days in media alone. Cell turnover and survival were measured by flow cytometry (Fig 6A). In the absence of stimulation, the majority of peritoneal cells died after 5 days of culture, with only ~20–30% of the input cells surviving. Knockout of IL-10 resulted in a significant reduction in the proportion of live cells to ~5–10% and appeared to particularly affect the B1a cell population in both B6 and B6.NZBc4m mice, as the proportion of these cells within the live cell population was reduced in the absence of IL-10 (Fig 6B and 6C). Interestingly, in B6.NZBc4m mice, neither the expansion of CD5^+^ B cells nor their enhanced turnover capacity as compared to B6 B cells was affected by the IL-10 knockout (Fig 6D). These findings suggest that IL-10 is required for peritoneal CD5^+^ B cell survival but has no effect on the proliferative capacity of these cells.

## Discussion

In this study, we have further dissected the immunogenetic basis for the expansion of innate-like lymphocytes and suppression of autoimmunity associated with the NZB c4 32 to 151 Mb interval. We show that the genetic locus leading to expansion of CD5^+^ B cell and NKT cell populations is localized to the mid 91 to 123 Mb region, whereas the predominant locus leading to suppression of autoimmunity is localized to the 123 to 151 Mb telomeric region. We also identify a critical role for IL-10 in restraining autoantibody production and supporting the expansion of innate-like lymphocytes.

Expansion of splenic and peritoneal CD5^+^ B cells is a well-documented feature of B6.*Sle2* congenic mice, which have an introgressed NZM2410 chromosome 4 interval [30,31]. The *Sle2* interval has a mixture of homozygous NZB- and NZW-derived genetic material. In contrast to our mice, which have entirely homozygous NZB regions, *Sle2* mice have an NZW interval that extends from 55 to 100 Mb and an NZB interval that extends from 100 to 128 Mb. Previous studies examining *Sle2* congenic mice have shown that the expansion of B1a and splenic CD5^+^ B cells localizes to the NZB interval and is likely the result of a genetic polymorphism leading to reduced levels of the Cdkn2c inhibitor, p18 [28,29,32]. The NZB c4m region, where we have localized the expansion of CD5^+^ B cells, overlaps with this interval and contains within it the p18 gene, providing support for these findings. However, we continue to document a previously unreported expansion of NK and NKT cells in B6.NZBc4 mice. This expansion is likely the result of another unidentified gene within the middle 91 to 123 Mb NZB-derived region, unique to our mice. Of note, Jak1, which lies downstream of IL-15, IL-22, and IL-7 signaling, is located at 101 Mb on chromosome 4 at the border of the *Sle2* NZB interval, and if functionally altered could result in increased homeostatic expansion of innate-like lymphocytes by promoting cytokine signaling [33,34].

Our previous work has shown that the expansion of IL-10-producing B cells correlates with disease suppression in bicongenic B6.NZBc1c4 mice and that transfer of B6.NZBc4 splenic B cells reduces the frequency of pro-inflammatory T cells in B6.NZBc1 lupus-prone recipient mice [7]. Additional adoptive transfer experiments provided support for a potential role of CD5^+^ B cells in this suppression [7]. Therefore, we anticipated that creation of bicongenic mice with a shorter telomeric chromosome 4 interval spanning 123 to 151 Mb, which lacked expansion of these cells, would result in little or no suppression. To our surprise, B6.NZBc1c4t maintained suppression of anti-ssDNA and anti-dsDNA antibodies, despite an absence of CD5^+^ B cell expansion. However, these mice did exhibit an increase in anti-chromatin IgG antibodies, as compared to mice with the longer NZB c4 congenic interval, suggesting that expansion of the CD5^+^ B cell compartment may lead to somewhat enhanced suppression. While these data indicate that expansion of CD5^+^ B cells is not required for suppression, they do not rule out a role for the CD5^+^ B cell population in this process, given our previous adoptive transfer results. It is possible that the CD5^+^ B cell population in B6.NZBc4t has altered function leading to enhanced suppressive activity. Along these lines, previous studies have shown that B6.*Sle2* mice have impaired receptor editing of nuclear antigen-reactive B cells [35,36]. It is possible that this leads to increased numbers of B cells with low affinity for nuclear antigens that are preferentially selected into the CD5^+^ regulatory B cell compartment. Alternatively, CD5^+^ B regulatory cells may not be the sole mechanism mediating suppression of autoimmunity in B6.NZBc1c4 bicongenic mice, as a genetic locus that leads to decreased autoantibody production in a graft vs host autoimmunity model has been identified in the NZB-derived *Sle2* region located between 115 to 128Mb [37,38]. This suppression has been shown to be mediated by non-lymphoid bone marrow-derived populations and may result from altered function of the G-CSFR.

In support of our hypothesis that CD5^+^ IL-10-producing B cells are important to disease suppression, knockout of IL-10 in B6.NZBc4 mice resulted in a breach of tolerance with enhanced production of IgM autoantibodies and an increase in MZ B cells. It is possible that the increase in MZ B cells could promote autoantibody production and autoimmunity in these mice, as shown by others [39–41]. It is also tempting to speculate that the production of autoantibodies in IL-10 knockout B6.NZBc4 mice arises from the presence of the previously identified *Sle2* receptor editing defect. In this context, loss of IL-10 in low affinity nuclear antigen-reactive B cells could convert these cells from a regulatory to a stimulatory phenotype. Alternatively, IL-10 may play a role in lineage commitment, with cells that are normally selected into the CD5^+^ B cell compartment being selected into the marginal zone compartment in the absence of IL-10, where they could then become activated to produce autoantibodies. As a caveat, it is important to remember that the ablation of IL-10 was a global knockout that could alter the function of numerous cells types including IL-10 producing Tregs, Tfregs, iNKT10 and others that could contribute to the overall phenotype. Although we believe from our previous work that CD5+ B cells are the most pertinent population in our model of disease, we have not ruled out the role of these other IL-10 dependent suppressive populations.

Surprisingly, loss of IL-10 also resulted in a number of unanticipated effects on the survival and/or selection of innate-like lymphocytes. In our congenic mice, knockout of IL-10 ablated the expansion of splenic and peritoneal CD5^+^ B cells as well as NKT cells. Previous work examining the impact of an IL-10 knockout on NZB mice showed no changes in peritoneal B1a cell numbers but greatly reduced development of “malignant” CD5^+^ B1 cells in the blood and spleen [42]. In another study, administration of anti-sense IL-10 oligonucleotide induced apoptosis and cell cycle disruption in malignant B1 clones from NZB mice, while increasing cyclin E, D2, and A, and reducing p27 levels [43]. Our data provides support for the role of IL-10 as a survival factor for CD5^+^ B cells, but suggests that for non-malignant CD5^+^ B cells, IL-10 does not appear to be required for cellular proliferation. Indeed, the enhanced turnover capacity of peritoneal B1a cells from B6.NZBc4m mice was retained despite the absence of IL-10. Although we have not directly examined the impact of IL-10 knockout on the survival of other innate-like lymphoid populations, it is likely that this cytokine plays a similar role for these cells, as the proportion of NK and NKT cells was also markedly reduced in the absence of IL-10.

In summary, we have localized the expansion of CD5^+^ B and NKT cells to the NZBc4m interval, identified a critical role for IL-10 in supporting this expansion, and in its absence, unmasked a minor breach in tolerance in B6.NZBc4 mice. Although the effect of these losses on autoimmunity was modest in B6.NZBc4 mice, other groups have shown that the gain or loss of IL-10 competent regulatory B or NKT cells can have profound effects on disease [10,44–52]. Our data suggests that IL-10 may act through multiple mechanisms to prevent the progression of autoimmunity and our work adds to this literature by highlighting the novel role it may play in supporting the expansions of suppressive populations.

## Materials and Methods

### Ethics Statement

Mice were housed in a Canadian Council on Animal Care approved facility at the Krembil Research Institute in the Krembil Discovery Tower, part of the University Health Network. All experiments performed in this study were approved by the Animal Care Committee of the University Health Network (Animal Use Protocol 123).

### Mice

B6 and NZB mice were purchased from Taconic (Germantown, NY) and Harlan Sprague Dawley (Blackthorn, England), respectively. Congenic mice were generated as previously described [6]. B6.IL-10-/- (B6.129P2-Il10tm1Cgn/J) mice were originally obtained from The Jackson Laboratory (Bar Harbor, ME) and bred onto the various congenic backgrounds using primer assisted breeding. Only female mice were used for experiments in this study, with littermate controls.

### Flow Cytometry

Splenocytes were harvested and RBC lysed, as previously described [6]. Briefly, half a million cells were incubated with mouse IgG (Sigma-Aldrich, St Louis, MO, USA) for 15 minutes on ice prior to staining with various combinations of directly conjugated mAbs for 30 minutes on ice. The following antibodies were used for primary staining (all purchased from BioLegend, San Diego, CA, USA or BD Biosciences, San Diego, CA, USA): FITC-conjugated anti-TCRβ(H57-597), -CD3ε(145-2C11), -CD23(B3B4); PE-conjugated anti-NK1.1(PK136), -CD24(M1/69), -CD5(53–7.3), -B220(RA3-6B2); PE-Cy7 anti-CD19(6D5), and -CD8(53–6.7); Allophycocyanin-conjugated anti-CD21(7E9), -CD19(6D5), -CD5(53–7.3), and -CD25(3C7); BV605 conjugated anti-B220(RA3-6B2) and -CD3ε(145-2C11); and Pacific Blue-conjugated anti-CD4(GK1.5) and -B220(RA3-6B2). Dead cells were identified by staining with 0.6ug/mL Propidium Iodide (Sigma Aldrich, St Louis, MO, USA). Allophycocyanin-conjugated unloaded and PBS-57–loaded mouse CD1d tetramers were generously provided by the National Institutes of Health Tetramer Core Facility (Atlanta, GA). Events were collected on a BD LSRII or BD FACSCanto and analyzed using FlowJo software (Tree Star Inc, Ashland, OR, USA).

### Detection of IL-10-producing B cells

IL-10 production by B cells was examined as previously described [53]. Briefly, 0.5 x 10^6^ RBC-depleted splenocytes were plated in 96 well flat bottom plates and stimulated for 4–5 hours with PMA (50ng/mL, Sigma-Aldrich, St Louis, MO, USA), Ionomycin (500ng/mL, 50ng/mL, Sigma-Aldrich, St Louis, MO, USA), and LPS (10ug/mL, Sigma Aldrich, E-coli 011:B4, St Louis, MO, USA) in the presence of GolgiStop (BD Biosciences, San Diego, CA, USA). Following stimulation, cells were stained with Near Infrared Live/Dead stain (Gibco, Waltham, MA, USA) and various conjugated antibodies directed against extracellular markers, and then fixed and permeabilized. The cells were then stained with allophycocyanin-conjugated anti-IL-10 (JES5-16E3, BioLegend, San Diego, CA, USA) for 30 minutes on ice.

### Autoantibody Measurements

Anti-chromatin, anti-ssDNA, and anti-dsDNA IgM, IgG, IgG1, and IgG2a autoantibodies, were measured by ELISA, as previously described [6]. Briefly, 96 well flat bottom plates were coated with antigen and left overnight. Serum was diluted 1 in 50 and added in triplicate to the plates. Bound antibodies were detected using alkaline-phosphatase conjugated anti-IgM, -IgG, -IgG1, -IgG2a, IgG2b, IgG2c, or IgG3 secondary reagents (Southern Biotech; Birmingham, AL). Substrate (4-nitrophenyl phosphate disodium salt hexahydrate, Sigma-Aldrich, St Louis, MO, USA) was added, and the OD of each well was measured at a wavelength of 405 nm. Values were standardized from plate to plate by running known B6 and NZB controls.

### CFSE Staining and Peritoneal B cell Culture

Peritoneal lavages were taken from mice previously injected with 5mL complete RPMI 1640 (Gibco, Waltham, MA, USA) media supplemented with 10% FBS (Wisent, ST-BRUNO, Quebec, Canada), L-glutamine (Gibco, Waltham, MA, USA), non-essential amino acids (Gibco, Waltham, MA, USA), and penicillin and streptomycin (Gibco, Waltham, MA, USA). Immediately after lavage, cells were transferred to T25 flasks (BD Biosciences, San Diego, CA, USA) and rested for 2 hours in a 37°C, 5% CO_2_ tissue culture incubator. Suspension cells were collected and washed with PBS prior to staining with CFSE (Gibco, Waltham, MA, USA). 10^5^ stained cells were left unstimulated in 96 well flat bottom culture plates (BD Biosciences, San Diego, CA, USA). After 5 days, cells were collected and stained as described above.

### Statistics

For comparisons of differences between three or more groups, a Kruskal-Wallis test was used followed by Dunns’ post-test for multiple comparisons. For comparison between wild-type and knockout animals, Mann-Whitney *U* tests were performed. All statistical analyses were performed using GraphPad software (La Jolla, CA, USA).

## Supporting Information

S1 Fig Total spleen and peritoneal cavity counts. Total cell numbers for peritoneal and splenic cells were counted using a hemocytometer. Total cell counts for mice used in Fig 1(A,B), Fig 2(C,D) and Fig 3-6(E,F). Each point represents a single mouse, with the lines for each group representing the median. Statistical analyses were carried out using a Mann-Whitney *U* tests.
(PDF)

S2 Fig Knockout of IL-10 is penetrant in B6, B6.NZBc4m, and B6.NZBc4 mice. Representative flow cytometry plot and results of IL-10 knockout in congenic animals. Splenocytes were stimulated for 4–5 hours with LPS, PMA, and Ionomycin in the presence of GolgiStop. IL-10 knockout was penetrant in all animals with a complete loss of cytokine production. Each point represents a single mouse, with the lines for each group representing the median.
(PDF)

S3 Fig Levels of IgG1 and IgG2a autoantibodies in IL-10 knockout mice are unchanged. Levels of anti-ssDNA, -dsDNA, and–chromatin were measured as previously described. Each point represents a single mouse, with the lines for each group representing the median.
(PDF)

S4 Fig The frequency of splenic regulatory T cells is unchanged in B6.NZBc4m IL-10 knockout mice. (A) Representative flow cytometry plot of CD25^+^Foxp3^+^ and Foxp3^+^ T regulatory cells from 4 month old B6 mice. (B) The frequency of splenic Foxp3^+^ T cells is unchanged in IL-10 knockout mice. (C) The frequency of CD25^+^ T regulatory cells was significantly increased in B6 IL-10 knockout mice but unchanged in congenic B6.NZBc4m mice regardless of IL-10 status. For Treg staining, RBC-depleted splenocytes were stained for extracellular markers, as described in materials and methods. After staining, cells were fixed and permeabilized with Foxp3 fixation/permeabilization buffer (Affymetrix, Santa Clara, CA, USA), washed, and stained with PE-conjugated anti-Foxp3 (FJK-16s, Affymetrix, Santa Clara, CA, USA). Each point represents a single mouse, with the lines for each group representing the median. Statistical analyses were carried out using a Mann-Whitney *U* test between homozygous and IL-10 knockout animals of the same genetic background. * P < 0.05, ** P < 0.01.
(PDF)
