# Supplementary material for: IL-10 Production Is Critical for Sustaining the Expansion of CD5+ B and NKT Cells and Restraining Autoantibody Production in Congenic Lupus-Prone Mice
Source: PLoS One. 2016 Mar 10;11(3):e0150515. doi: 10.1371/journal.pone.0150515 (PMC4786215; doi:10.1371/journal.pone.0150515)
Supplement: S1 Fig — Total cell numbers for peritoneal and splenic cells were counted using a hemocytometer. Total cell counts for mice used in Fig 1(A,B), Fig 2(C,D) and Fig 3-6(E,F). Each point represents a single mouse, with the lines for each group representing the median. Statistical analyses were carried out using a Mann-Whitney U tests. (PDF) [file pone.0150515.s001.pdf]

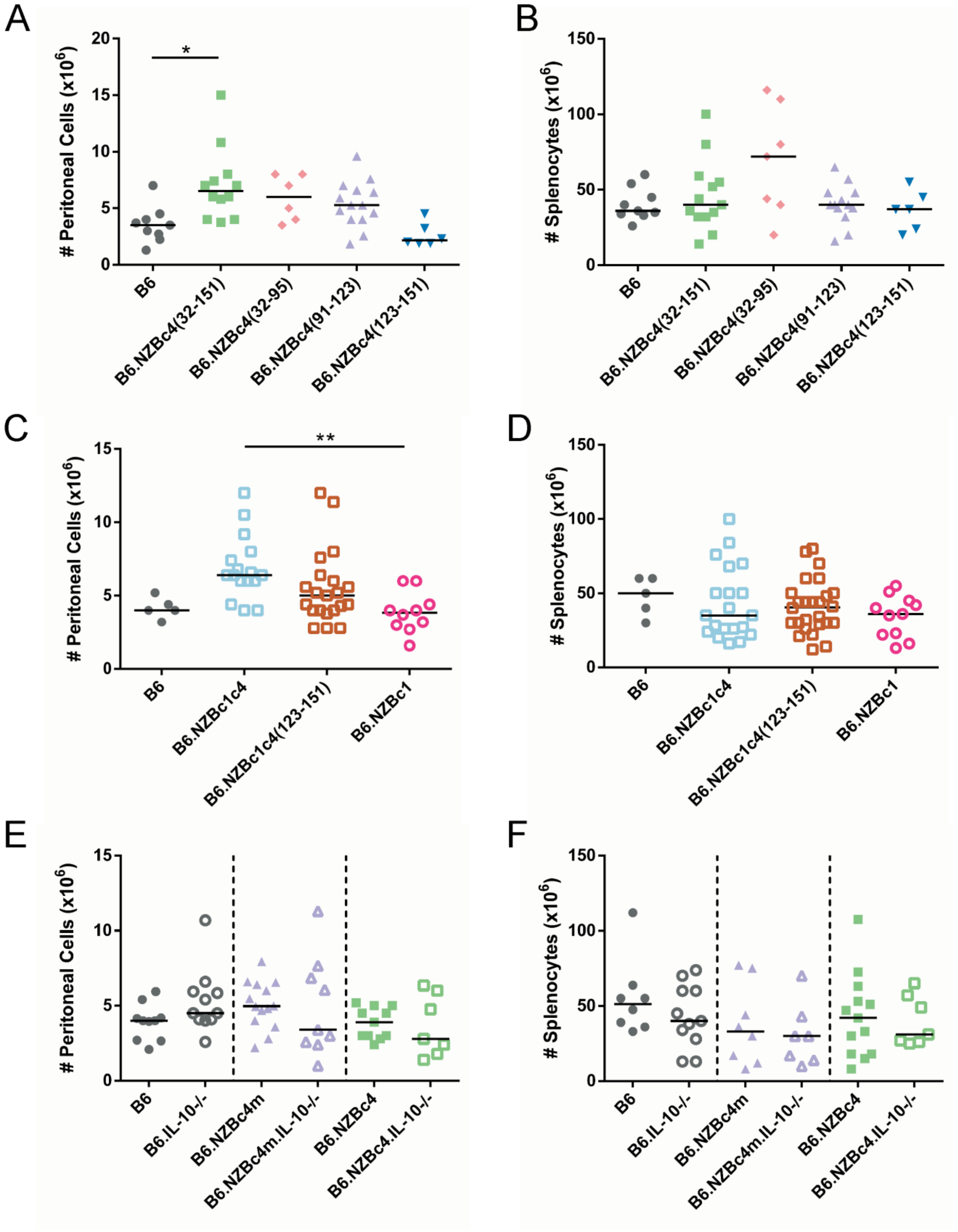

S1 Fig. Total spleen and peritoneal cavity counts. Total cell numbers for peritoneal and splenic cells were counted using a hemocytometer. Total cell counts for mice used in Figure 1(A,B), Figure 2(C,D) and Figures 3-6(E,F). Each point represents a single mouse, with the lines for each group representing the median. Statistical analyses were carried out using a Mann-Whitney U tests.
