# Supplementary material for: IL-10 Production Is Critical for Sustaining the Expansion of CD5+ B and NKT Cells and Restraining Autoantibody Production in Congenic Lupus-Prone Mice
Source: PLoS One. 2016 Mar 10;11(3):e0150515. doi: 10.1371/journal.pone.0150515 (PMC4786215; doi:10.1371/journal.pone.0150515)
Supplement: S2 Fig — Representative flow cytometry plot and results of IL-10 knockout in congenic animals. Splenocytes were stimulated for 4–5 hours with LPS, PMA, and Ionomycin in the presence of GolgiStop. IL-10 knockout was penetrant in all animals with a complete loss of cytokine production. Each point represents a single mouse, with the lines for each group representing the median. (PDF) [file pone.0150515.s002.pdf]

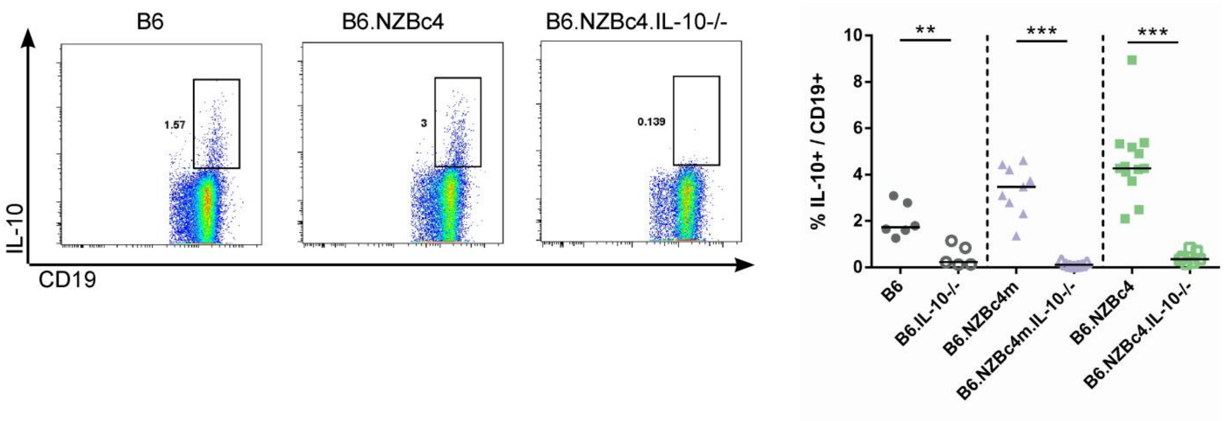

S2 Fig. Knockout of IL-10 is penetrant in B6, B6.NZBc4m, and B6.NZBc4 mice. Representative flow cytometry plot and results of IL-10 knockout in congenic animals. Splenocytes were stimulated for 4-5 hours with LPS, PMA, and Ionomycin in the presence of GolgiStop. IL-10 knockout was penetrant in all animals with a complete loss of cytokine production. Each point represents a single mouse, with the lines for each group representing the median.
