# Supplementary material for: IL-10 Production Is Critical for Sustaining the Expansion of CD5+ B and NKT Cells and Restraining Autoantibody Production in Congenic Lupus-Prone Mice
Source: PLoS One. 2016 Mar 10;11(3):e0150515. doi: 10.1371/journal.pone.0150515 (PMC4786215; doi:10.1371/journal.pone.0150515)
Supplement: S3 Fig — Levels of anti-ssDNA, -dsDNA, and–chromatin were measured as previously described. Each point represents a single mouse, with the lines for each group representing the median. (PDF) [file pone.0150515.s003.pdf]

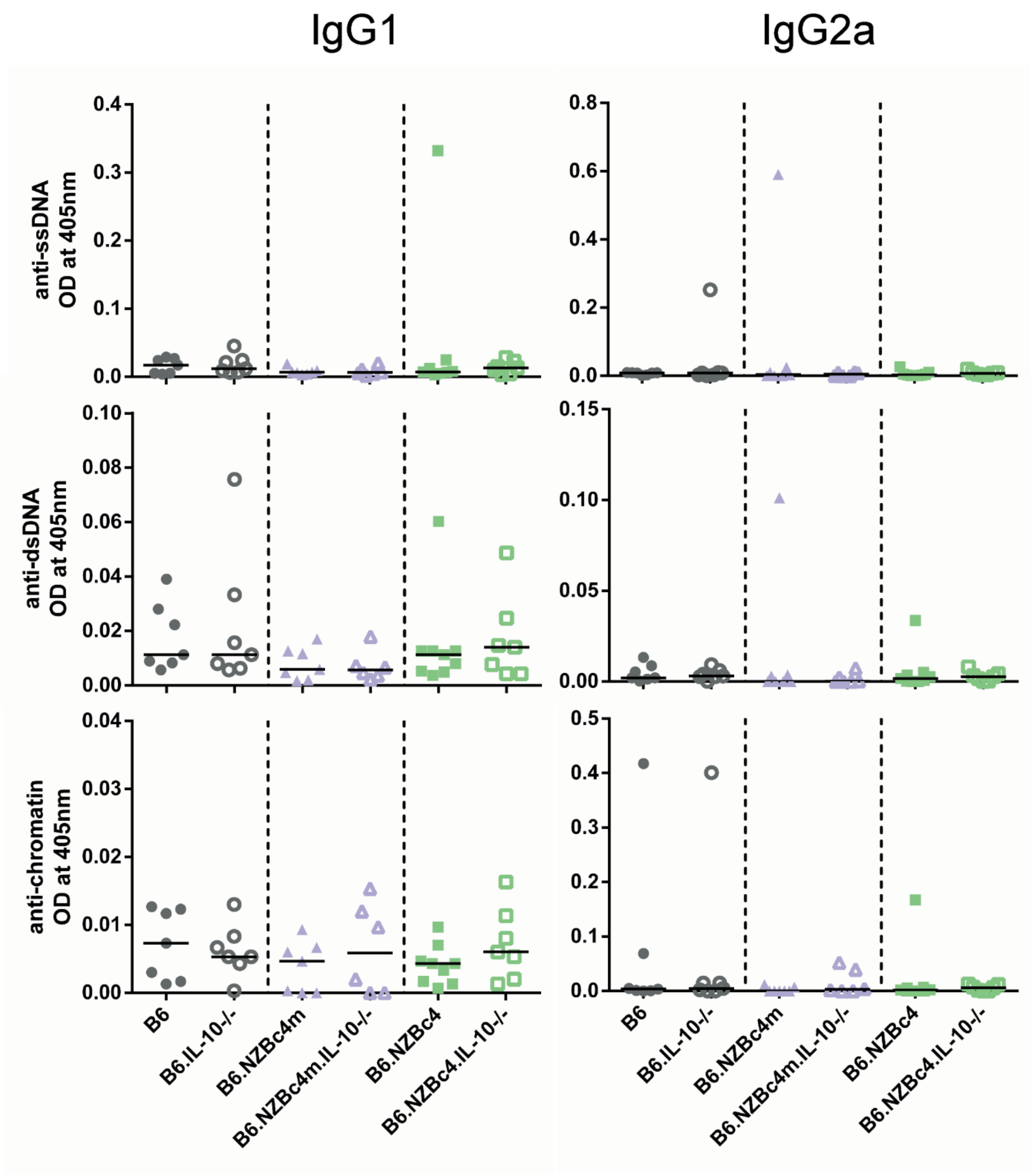

S3 Fig. Levels of IgG1 and IgG2a autoantibodies in IL-10 knockout mice are unchanged. Levels of anti-ssDNA, -dsDNA, and –chromatin were measured as previously described. Each point represents a single mouse, with the lines for each group representing the median.
