# Supplementary material for: IL-10 Production Is Critical for Sustaining the Expansion of CD5+ B and NKT Cells and Restraining Autoantibody Production in Congenic Lupus-Prone Mice
Source: PLoS One. 2016 Mar 10;11(3):e0150515. doi: 10.1371/journal.pone.0150515 (PMC4786215; doi:10.1371/journal.pone.0150515)
Supplement: S4 Fig — (A) Representative flow cytometry plot of CD25+Foxp3+ and Foxp3+ T regulatory cells from 4 month old B6 mice. (B) The frequency of splenic Foxp3+ T cells is unchanged in IL-10 knockout mice. (C) The frequency of CD25+ T regulatory cells was significantly increased in B6 IL-10 knockout mice but unchanged in congenic B6.NZBc4m mice regardless of IL-10 status. For Treg staining, RBC-depleted splenocytes were stained for extracellular markers, as described in materials and methods. After staining, cells were fixed and permeabilized with Foxp3 fixation/permeabilization buffer (Affymetrix, Santa Clara, CA, USA), washed, and stained with PE-conjugated anti-Foxp3 (FJK-16s, Affymetrix, Santa Clara, CA, USA). Each point represents a single mouse, with the lines for each group representing the median. Statistical analyses were carried out using a Mann-Whitney U test between homozygous and IL-10 knockout animals of the same genetic background. * P < 0.05, ** P < 0.01. (PDF) [file pone.0150515.s004.pdf]

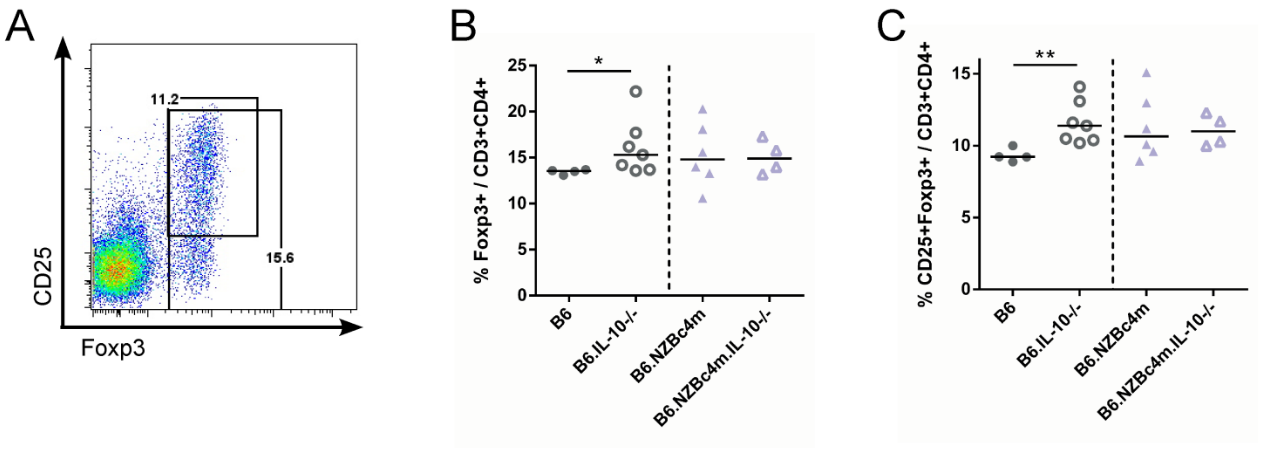

S4 Fig. The frequency of splenic regulatory T cells is unchanged in B6.NZBc4m IL-10 knockout mice. (A) Representative flow cytometry plot of CD25+Foxp3+ and Foxp3+ T regulatory cells from 4 month old B6 mice. (B) The frequency of splenic Foxp3+ T cells is unchanged in IL-10 knockout mice. (C) The frequency of CD25+ T regulatory cells was significantly increased in B6 IL-10 knockout mice but unchanged in congenic B6.NZBc4m mice regardless of IL-10 status. Each point represents a single mouse, with the lines for each group representing the median. Statistical analyses were carried out using a Mann-Whitney U test between homozygous and IL-10 knockout animals of the same genetic background. * $P < 0.05$, ** $P < 0.01$.
